# Shikonin protects against D-Galactosamine and lipopolysaccharide-induced acute hepatic injury by inhibiting TLR4 signaling pathway

**DOI:** 10.18632/oncotarget.21070

**Published:** 2017-09-16

**Authors:** Meng-Xiang Lin, Yong-Xiang Yi, Pei-Pei Fang, Shan-Shan Huang, Chen-Wei Pan, Ling-Xiang Jin, Tong Zhang, Guang-Yao Zhou

**Affiliations:** ^1^ Department of Anesthesiology, Critical Care and Pain Medicine, The Second Affiliated Hospital and Yuying Children’s Hospital of Wenzhou Medical University, Wenzhou, Zhejiang, 325027, P.R. China; ^2^ Department of Hepatobiliary Surgery, The Second Affiliated Hospital of Southeast University, Nanjing, Jiangsu, 210003, P.R. China; ^3^ Department of Infectious Disease, The Second Affiliated Hospital and Yuying Children’s Hospital of Wenzhou Medical University, Wenzhou, Zhejiang, 325027, P.R. China; ^4^ Department of Hepatobiliary Surgery, The People’s Hospital of Xinghua, Jiangsu, 225700, P.R. China

**Keywords:** shikonin, LPS, hepatic injury, TLR4

## Abstract

Shikonin, a naphthoquinone isolated from the root of medical herb *Lithospermum erythrorhizon,* has been reported to have anti-inflammatory effect. However, there is no related research for the treatment of shikonin on hepaic injury. The purpose of this study was to investigate the effects of shikonin on D-Galactosamine and Lipopolysaccharide-induced hepatic injury in mice. Male BALB/c mice were pretreated with shikonin 1 h before LPS/D-GalN treatment. The pathological changes of hepatic injury were detected by H&E staining. The levels of TNF-α and IL-1β in hepatic tissues were detected by ELISA. The levels of alanine aminotransferase (ALT) and aspartate aminotransferase (AST) were also measured in this study. In addition, the expression of TLR4 and NF-κB were determined by western blot analysis. These results suggest that shikonin effectively prevents LPS/D-GalN-induced liver injury by inhibiting AST and ALT levels, as well as inflammatory cytokines TNF-α and IL-1β production. The expression of TLR4 and NF-κB activation induced by LPS/D-GalN were also inhibited by treatment of shikonin. *In vitro*, shikonin significantly inhibited LPS-induced TNF-α and IL-1β production, as well as TLR4 expression and NF-κB activation. In conclusion, the results of the present study suggest that shikonin attenuates LPS/D-GalN-induced hepatic injury by inhibiting TLR4 signaling pathway.

## INTRODUCTION

Shikonin, a naphthoquinone isolated from the root of medical herb *Lithospermum erythrorhizon,* has been reported to have anti-inflammatory effect [1, 2]. A previous study showed that shikonin inhibited LPS-induced NO production in RAW264.7 cells [3]. Shikonin also inhibited LPS-induced TNF-α release in rat primary macrophage [1]. Shikonin has been known to attenuate inflammatory mediator production in BV2 microglial cells [4]. Furthermore, shikonin has been reported to attenuate LPS-induced acute lung injury in mice [5]. In addition, shikonin was found to protect against experimental ischemic stroke by inhibiting TLR4 signaling pathway [6]. However, the effects of Shikonin on LPS/GalN-induced acute liver injury remain unclear. The purpose of this study was to investigate the protective effects and mechanisms of shikonin on LPS/GalN-induced hepatic injury.

Fulminant hepatic failure (FHF) is an inflammatory disease that often leads to hepatocellular apoptosis and hepatic injury [7, 8]. Till now, there is no specific therapy for FHF except for liver transplantation [9]. Previous studies showed that a variety of factors that could lead to hepatic injury, such as toxic insult, drugs, and virus infection [10–12]. LPS is a major component of the cell membrane of *Gram-negative* bacteria. Studies showed that LPS could activate TLR4 signaling pathway in kupffer cells, which lead to the release of inflammatory cytokines [13, 14]. These inflammatory cytokines has the ability to exacerbate liver injury [15]. GalN has the ability to extend LPS-induced liver injury [16]. LPS and D-GalN-induced hepatic injury in mice model is similar to acute hepatic injury in the clinical setting [17]. Oxidative stress also plays a critical role in the development of liver injury [18]. Inhibition of inflammatory response and oxidative stress could attenuate LPS/D-GalN-induced liver injury [19]. Therefore, in the present study, we used LPS and D-GalN-induced hepatic injury model to investigate the protective effects and mechanism of shikonin on hepatic injury.

## RESULTS

### Shikonin inhibits LPS/GalN-induced ALT and AST levels

**Figure 1:**
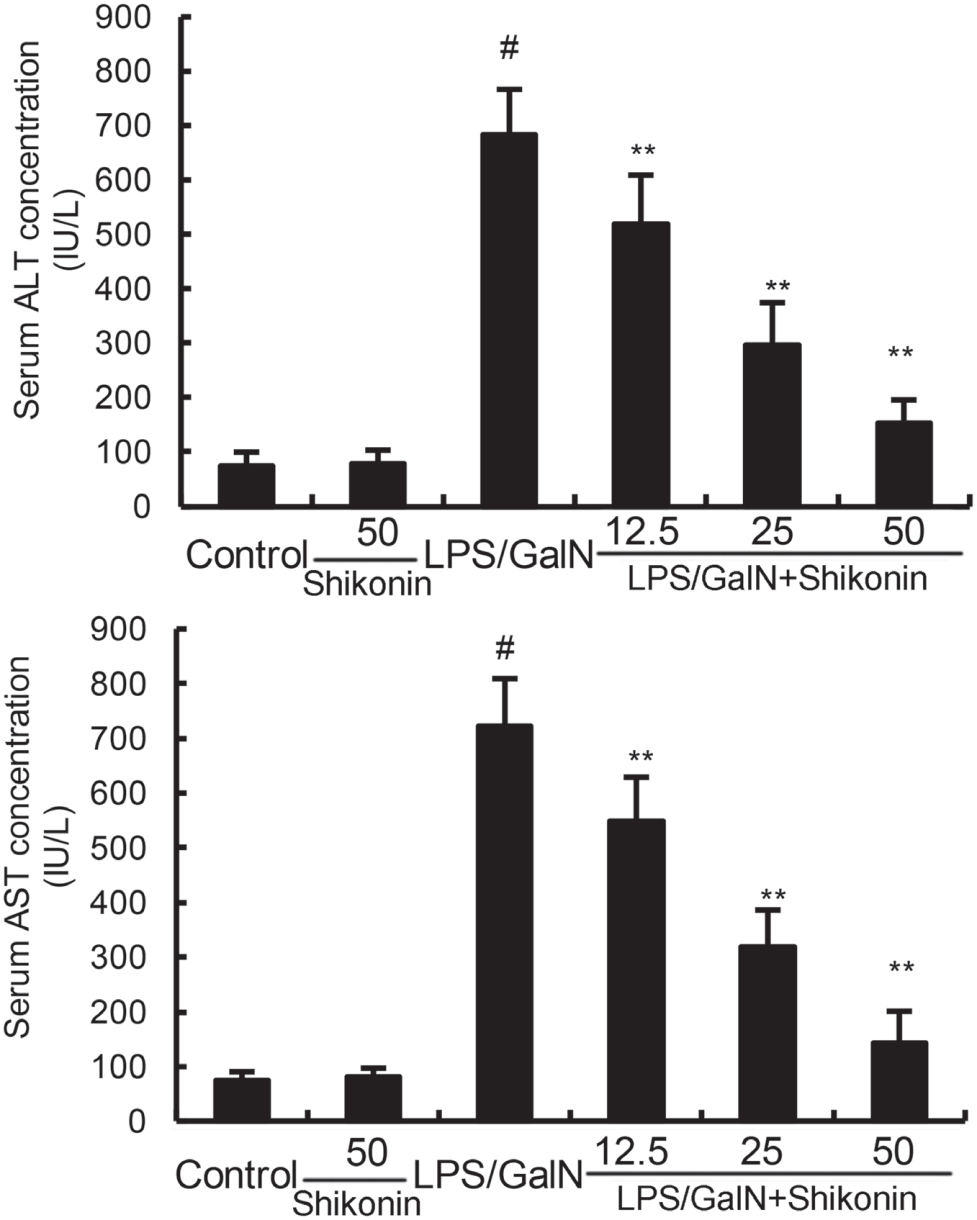
Effects of shikonin on serum ALT and AST levels The values presented are the mean ± S.E.M. of three independent experiments. P^#^<0.01 vs. control group, P*<0.05, P**<0.01 vs. LPS/GalN group.

The levels of ALT and AST were detected in this study. The results showed that shikonin alone did not affect the levels of ALT and AST. The levels of ALT and AST increased significantly in LPS/GalN group when compared with the control group. However, LPS/GalN-induced T ALT and AST production were dose-dependently inhibited by treatment of shikonin (Figure 1).

### Effects of shikonin on LPS/GalN-induced liver histopathologic changes

**Figure 2:**
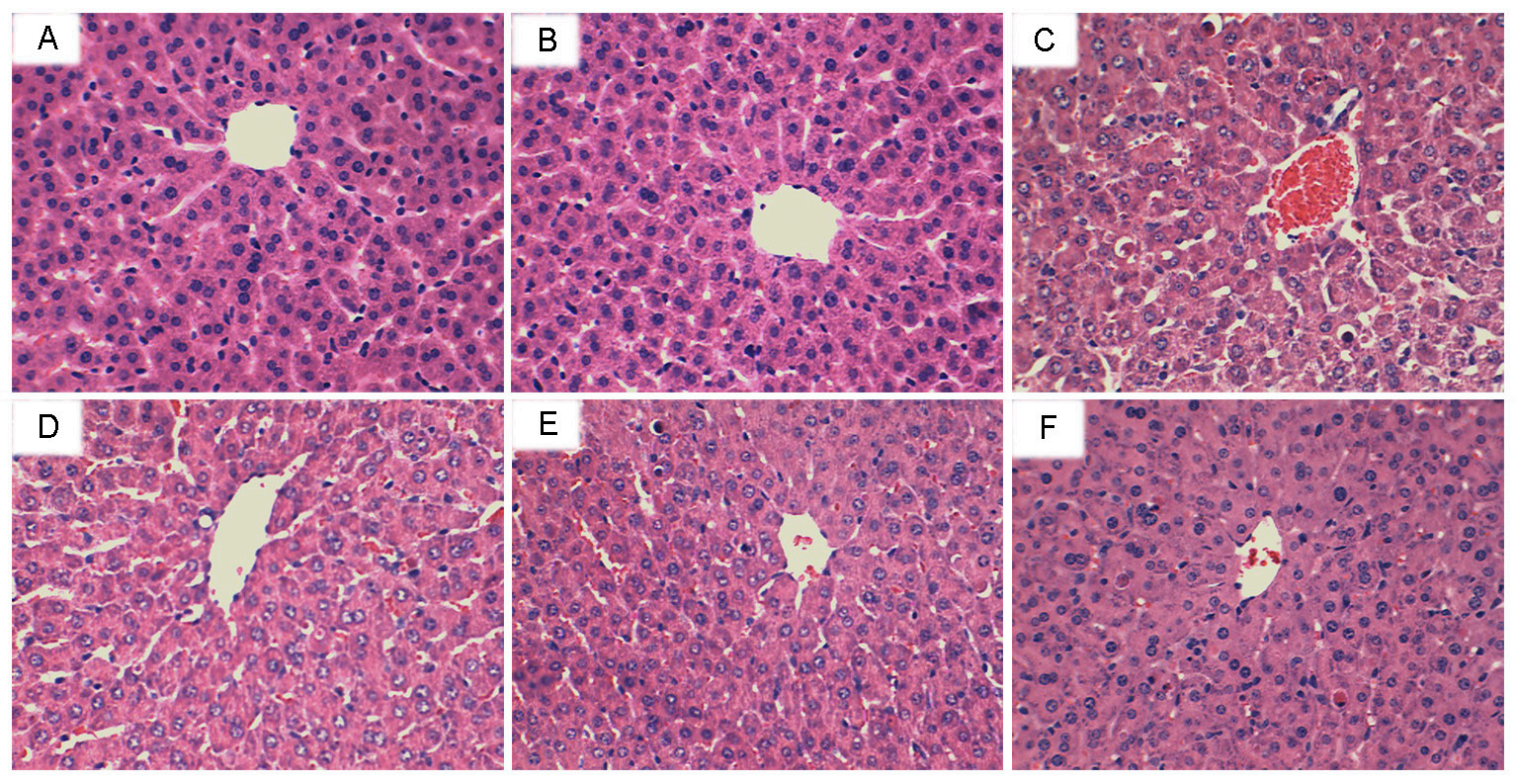
Effects of shikonin on histopathological changes in liver tissues Representative histological changes of liver obtained from mice of different groups. **(A)** Control group, **(B)** shikonin (50 mg/kg) group, **(C)** LPS/GalN group, **(D)** LPS/GalN + shikonin (12.5 mg/kg) group, **(E)** LPS/GalN + shikonin (25 mg/kg) group, **(F)** LPS/GalN + shikonin (50 mg/kg) group (Hematoxylin and eosin staining, magnification 200×).

To assess the protective effects of shikonin on LPS/GalN-induced liver injury, histological changes in the liver were detected by H&E staining. The results showed that no histopathologic changes were observed in liver tissues of the control group and shikonin alone group. Liver tissues of the LPS/D-GalN group showed severe histopathologic changes, including extensive hemorrhage, necrosis and neutrophil infiltration (Figure 2B). However, treatment of shikonin dose-dependently inhibited LPS/D-GalN-induced liver histopathologic changes (Figure 2C, D, E).

### Effects of shikonin on LPS/GalN-induced MPO activity, MDA and GSH production

**Figure 3:**
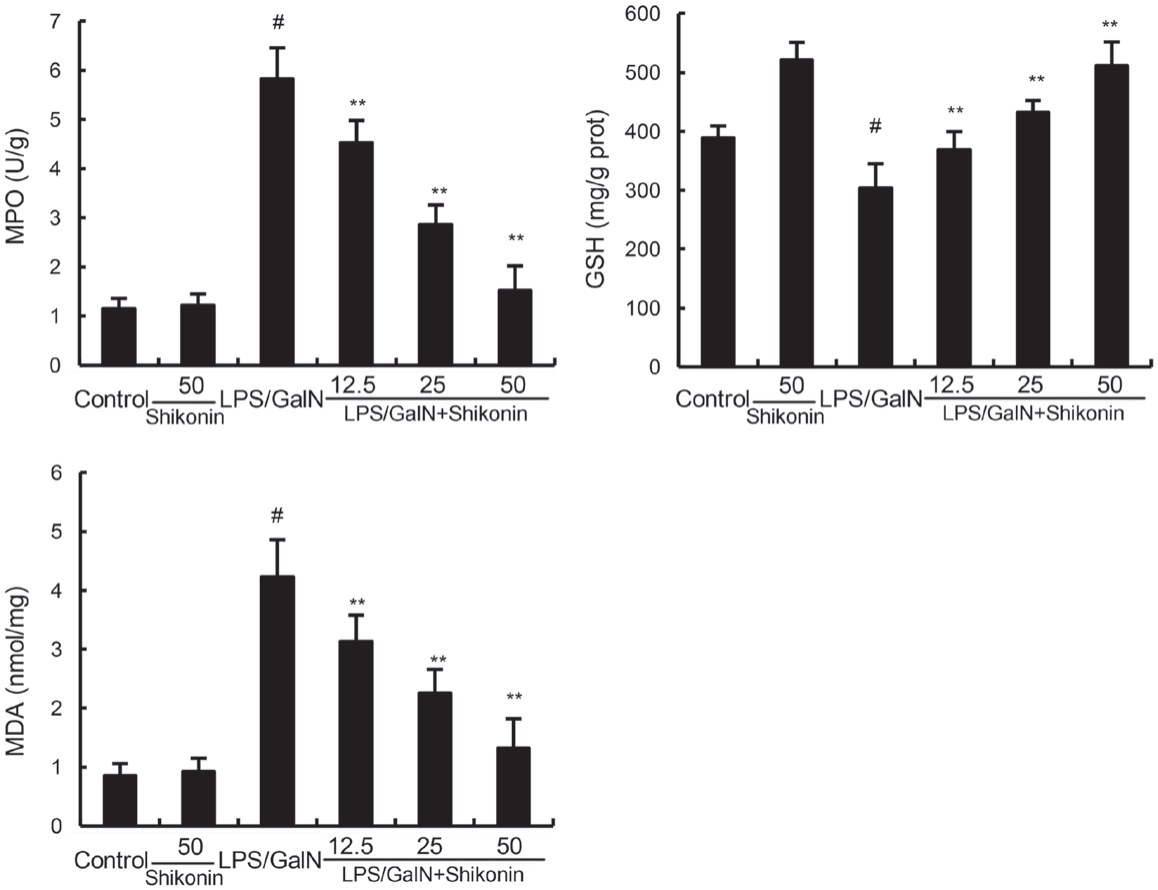
Effects of shikonin on liver MPO, MDA, and GSH levels The values presented are the mean ± S.E.M. of three independent experiments. P^#^<0.01 vs. control group, P*<0.05, P**<0.01 vs. LPS/GalN group.

MPO, a quantitative marker of neutrophil infiltration, was used to assess the infiltration of neutrophil in liver tissues [20]. The effects of shikonin on LPS/GalN-induced MPO activity were measured in this study. As shown in Figure 3, shikonin alone did not affect the activity of MPO. Compared with the control group, the MPO activity of LPS/GalN group exhibited significantly increased in liver tissues. However, this increase was inhibited with the administration of shikonin (Figure 3). Liver MDA content was used to assess lipid peroxidation in the liver. In this study, our results showed that shikonin significantly inhibited LPS/GalN-induced MDA production. Furthermore, LPS/D-GalN decreased the level of GSH, and the decrease was raised by shikonin treatment (Figure 3).

### Effects of shikonin on LPS/GalN-induced TNF-α and IL-1β production

**Figure 4:**
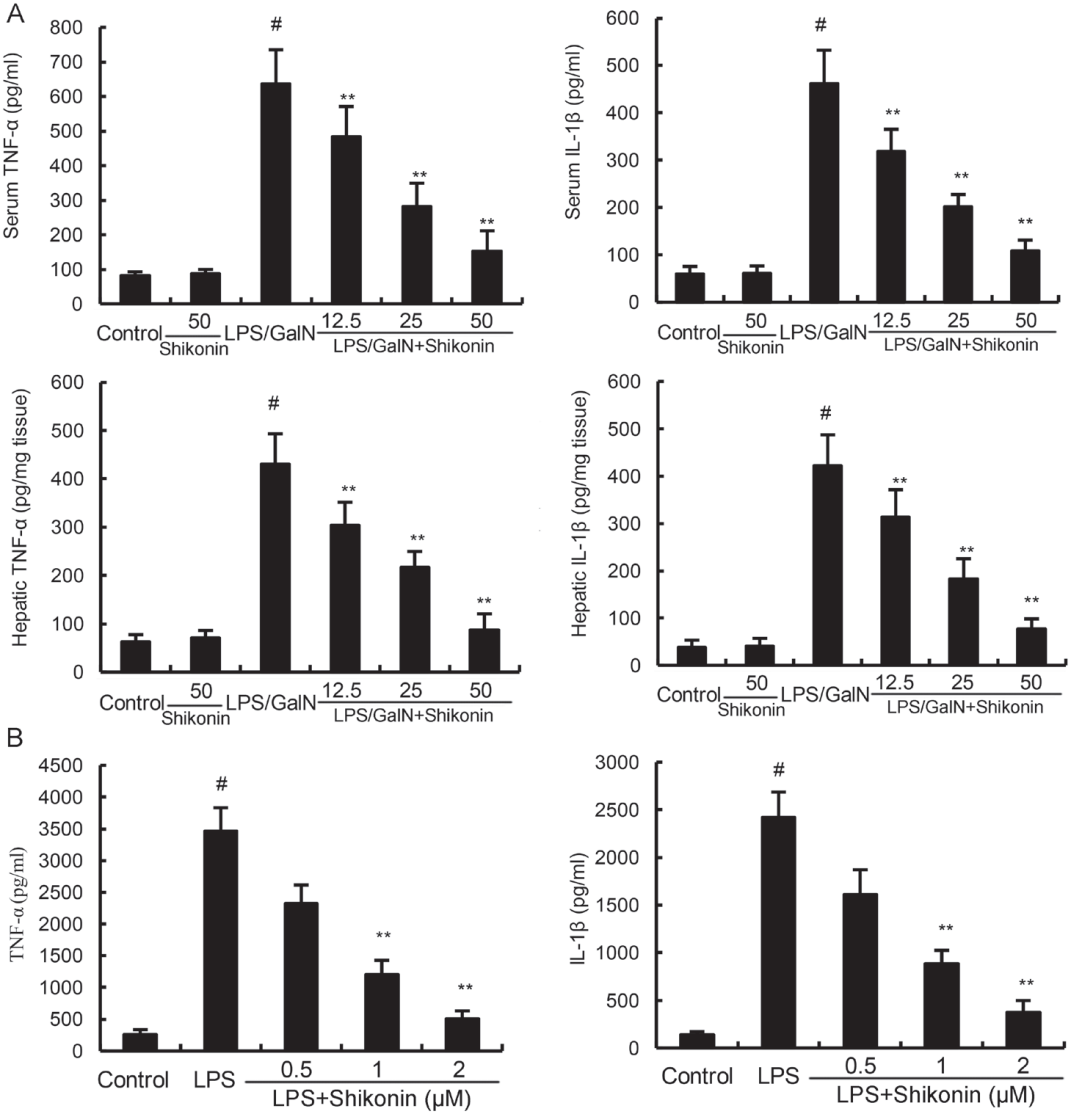
**(A)** Effects of shikonin on serum and hepatic TNF-α and IL-1β levels. **(B)** Effects of shikonin on TNF-α and IL-1β production in LPS-stimulated RAW264.7 cells. The values presented are the means ±S.E.M. of three independent experiments. P^#^<0.01 vs. control group, P*<0.05, P**<0.01 vs. LPS/GalN group.

The levels of TNF-α and IL-1β were detected by ELISA in this study. The results showed that shikonin alone did not affect the levels of TNF-α and IL-1β. The levels of TNF-α and IL-1β increased significantly in LPS/GalN group when compared with the control group. However, LPS/GalN-induced TNF-α and IL-1β production were dose-dependently inhibited by treatment of shikonin (Figure 4A). *In vitro*, shikonin significantly inhibited LPS-induced TNF-α and IL-1β production in RAW264.7 cells (Figure 4B).

### Effects of shikonin on LPS/GalN-induced TLR4 expression and NF-κB activation

**Figure 5:**
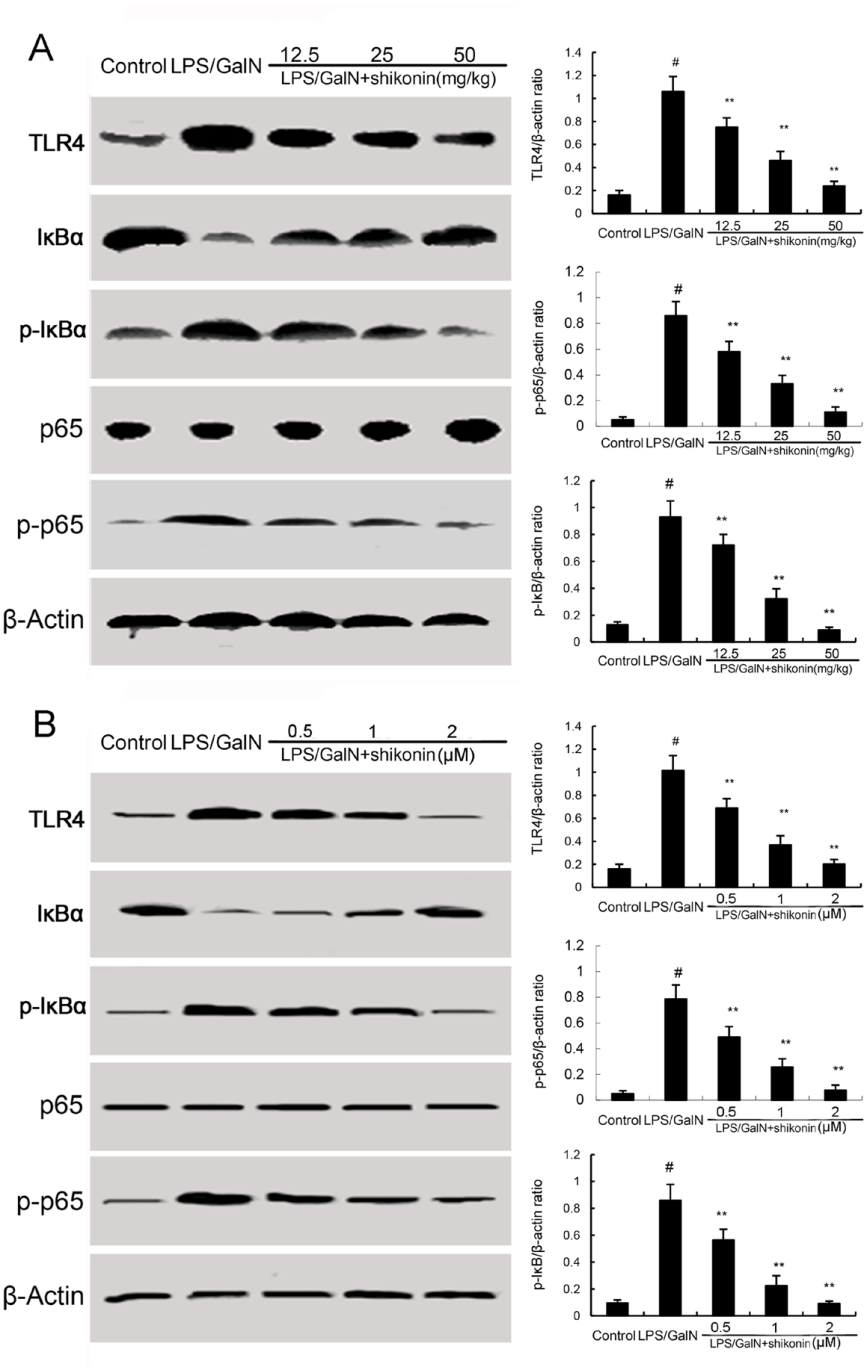
**(A)** Effects of shikonin on TLR4 expression and NF-κB activation in liver tissues. **(B)** Effects of shikonin on TLR4 expression and NF-κB activation in LPS-stimulated RAW264.7 cells. The values presented are the mean ±S.E.M. of three independent experiments. The density values of the Western blot were normalized for β-actin. P^#^<0.01 vs. control group, P*<0.05, P**<0.01 vs. LPS/GalN group.

**Figure 6:**
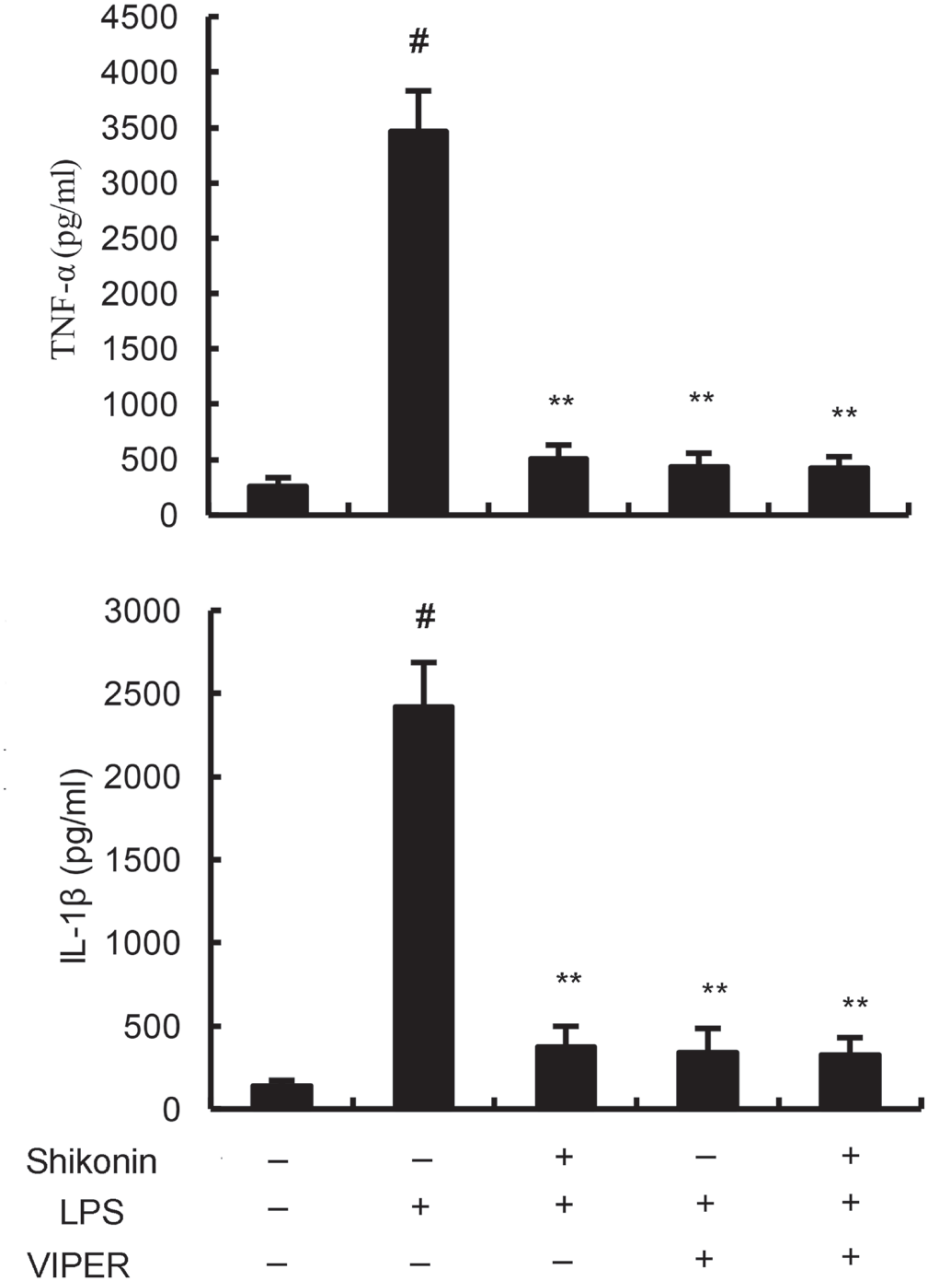
Effects of TLR4 inhibitor VIPER and shikonin on TNF-α and IL-1β production in LPS-stimulated RAW264.7 cells The values presented are the means ±S.E.M. of three independent experiments. P^#^<0.01 vs. control group, P*<0.05, P**<0.01 vs. LPS/GalN group.

TLR4 has been reported to play critical role in inflammatory response. To investigate the anti-inflammatory mechanism of shikonin, the effects of shikonin on TLR4 expression and NF-κB activation were detected in this study. The results showed that LPS/GalN significantly up-regulated the expression of TLR4, as well as the levels of phosphorylation of NF-κB and IκBα. However, LPS/GalN-induced TLR4 expression and NF-κB activation were significantly inhibited by treatment of shikonin (Figure 5A). *In vitro*, shikonin significantly inhibited LPS-induced TLR4 expression and NF-κB activation in RAW264.7 cells (5B). Furthermore, our results showed that the inhibitory effects of shikonin on TNF-α and IL-1β production were similar to TLR4 inhibitor VIPER (Figure 6).

## DISCUSSION

In our present study, we used LPS/GalN-induced mice liver injury model to investigate the protective effects and mechanism of shikonin on liver injury. We demonstrated that shikonin significantly inhibited LPS/GalN-induced ALT and AST production, as well as TNF-α and IL-1β production. Shikonin protected against LPS/GalN-induced liver injury by inhibiting TLR4 signaling pathway.

LPS/GalN-induced mice liver injury model has been widely used to evaluate the biological activities of hepatoprotective agents [21]. In this study, we evaluated the protective effects of shikonin on liver injury using this model. The histological observation of liver tissues showed that shikonin significantly attenuated LPS/GalN-induced liver injury. Serum ALT and AST are important markers of liver injury [22, 23]. In this study, our results showed that shikonin dose-dependently inhibited LPS/GalN-induced ALT and AST production. The results were consistent with the H&E staining and suggested that shikonin had protective effects against LPS/GalN-induced liver injury. Inflammation plays a critical role in the development of liver injury [15, 24]. LPS significantly induces the production of inflammatory cytokines TNF-α and IL-1β. And these cytokines lead to liver injury [25]. Studies showed that knock-out of TNF-α receptor completely abrogated LPS/GalN-induced liver injury. Furthermore, many compounds prevented LPS/GalN-induced liver injury by inhibiting TNF-α and IL-1β production [26, 27]. In the present study, our results showed that shikonin significantly inhibited LPS/GalN-induced TNF-α and IL-1β production. These results suggest that shikonin protects against LPS/GalN-induced liver injury by inhibiting inflammatory response. MDA, the end product of lipid peroxidation, often used to assess the oxidative stress of different tissues [28]. Our results showed that shikonin significantly inhibited LPS/GalN-induced MDA level. The results suggest that shikonin protects against LPS/GalN-induced liver injury by inhibiting oxidative stress.

LPS is the major component of *Gram-negative* bacteria. In LPS/GalN-induced liver injury model, LPS could induce the production of inflammatory cytokines in macrophages/Kupffer cells through the TLR4 receptor [29]. Studies showed that activation of TLR4 receptor by LPS could induce the activation of NF-κB, which subsequently lead to the production of inflammatory cytokines, such as TNF-α and IL-1β [30]. In addition, previous studies showed that TLR4 played an important role in liver injury [31]. TLR4 is involved in the mechanism of alcohol-induced liver injury and endotoxin-induced liver injury [32, 33]. Furthermore, studies showed that inhibition of TLR4 signaling pathway could attenuate LPS/GalN-induced liver injury [34]. In this study, our results showed that shikonin dose-dependently inhibited LPS/GalN-induced TLR4 expression and NF-κB activation. The results suggested that shikonin protected against LPS/GalN-induced liver injury by inhibiting TLR4 signaling pathway.

In conclusion, our results suggested that shikonin protected against LPS/GalN-induced acute liver injury by inhibiting of inflammation, which may be mediated by inhibition of TLR4/NF-κB signaling pathway. Shikonin may be a hopeful drug for prevention of acute liver injury.

## MATERIALS AND METHODS

### Reagents

Shikonin (purity>98%) was purchased from the Chinese drug administration and Biological Product Control (Beijing, China). Dimethyl sulphoxide (DMSO), LPS (Escherichiacoli, O55:B5) and D-galactosamine were purchased from Sigma-Aldrich (St. Louis, MO, USA). TNF-α and IL-1β ELISA kits were purchased from Pierce Biotechnology, Inc. (Rockford, IL, USA). The myeloperoxidase (MPO) determination kit, aspartate aminotransferase (AST), and alanine aminotransferase (ALT) detection kits were provided by the JianCheng Bioengineering Institute of Nanjing (Nanjing, China). Antibodies against TLR4, NF-κB p65, NF-κB p-p65, IκBα, p-IκBα, and β-actin were purchased from Cell Signaling Technology (Danvers, MA, USA). All other reagents were of analytical grade.

### Animals and experimental groups

Male BALB/c mice (6 weeks old) were obtained from the Center of Experimental Animals of Wenzhou Medical University (Wenzhou, China). The mice were housed in an animal room and allowed with food and water ad libitum. The temperature of the animal house was 24 ± 1 °C.

All animal studies were conducted according to the experimental practices and standards approved by the Animal Welfare and Research Ethics Committee at Wenzhou Medical University. Seventy-two mice were assigned to six groups: control group, Shikonin (50 mg/kg) alone group, LPS/GalN group, LPS/GalN+ Shikonin (12.5, 25, 50 mg/kg) groups. The mice of LPS/GalN group were injected intraperitoneally with LPS (60 mg/kg) and D-GalN (800 mg/kg). Shikonin (20 mg) was dissolved in dimethyl sulfoxide (DMSO, 200μl) and then further dissolved in PBS. Shikonin was administrated by intraperitoneal injection. The mice of LPS/GalN+ Shikonin (12.5, 25, 50 mg/kg) groups were received with Shikonin (12.5, 25, 50 mg/kg) 1 h after LPS/GalN challenge. 8 h after LPS/GalN treatment, the blood and liver tissues were collected for subsequent analysis. The chose of 8 h was based on previous studies [35].

### Haematoxylin and eosin (HE) staining

Liver tissues were collected, fixed in 10% buffered paraformaldehyde, dehydrated with graded alcohol and embedded in paraffin. Then the tissues were cut into 5 μm sections and the sections were stained with hematoxylin and eosin. The histopathologic changes were detected using an optical microscope (Olympus Optical Co, Tokyo, Japan).

### ALT and AST assays

Plasma samples were collected from the mice 8 h after the LPS/GalN injection. Serum levels of ALT and AST were detected by using test kits (Jiancheng Bioengineering Institute of Nanjing) according to the manufacturer’s protocols.

### MPO, MDA, and GSH assay

The liver tissues were collected 8 h after LPS/GalN treatment. Liver tissues were homogenized and centrifuged to acquire the supernatants. MPO activity in supernatant of liver tissues was measured by using commercial kit (Jiancheng Bioengineering Institute of Nanjing, Nanjing, China) according to the manufacturer’s protocols. The levels of MDA and GSH were measured by using the commercial detection kit (Nanjing Jiancheng Bioengineering Institute, Nanjing, China) according to the manufacturer’s instructions.

### ELISA assay

The liver tissues were collected 8 h after LPS/GalN treatment. The levels of inflammatory cytokines TNF-α and IL-1β were assayed using commercial ELISA kits (Pierce Biotechnology, Inc., Rockford, IL, USA) the manufacturer's protocols.

### Western blot analysis

Total proteins from liver tissues were extracted by using a protein extract kit (Shenggong, Shanghai, China) according to the manufacturer’s protocol. The proteins (30 mg) were fractionated by SDS-PAGE and transferred to PVDF membranes. The membranes were blocked with 5% (w/v) fat-free milk for 2h at room temperature. Then the membranes were probed with primary antibodies and horseradish peroxidase-conjugated secondary antibody. Antibody binding were visualized with the ECLPlus Western Blotting Detection System (GE Healthcare, Chalfont St Giles, UK).

### *In vitro* study

RAW264.7 cells were obtained from ATCC (USA). The cells were maintained in DMEM with 10% heat-inactivated FBS, penicillin (100 U/ml), and streptomycin (100 mg/ml) at 37°C in a humidified incubator under 5% CO_2_. The cells were treated with shikonin (0.5, 1, 2μM) 1 h before LPS treatment. For TLR4 inhibitory experiment, the cells were treated with a selective TLR4 inhibitor VIPER (Imgenex, San Diego, CA, USA) for 1 h and then treated with shikonin (2μM) and LPS. 24 h later, the levels of TNF-α and IL-1β were detected by ELISA.

### Statistical analysis

All data are expressed as the mean ± SEM. The significance of difference was assessed by one-way ANOVA followed by a Student-Newman-Keuls test. Statistical significance was accepted at *p* < 0.05.
